# Patient expectations for management of chronic non‐cancer pain: A systematic review

**DOI:** 10.1111/hex.12527

**Published:** 2016-12-23

**Authors:** Jose W. Geurts, Paul C. Willems, Craig Lockwood, Maarten van Kleef, Jos Kleijnen, Carmen Dirksen

**Affiliations:** ^1^ Department of Anaesthesiology and Pain Medicine Maastricht University Medical Centre Maastricht The Netherlands; ^2^ Department of Orthopaedic Surgery Maastricht University Medical Centre Maastricht The Netherlands; ^3^ The Joanna Briggs Institute The University of Adelaide Adelaide SA Australia; ^4^ School for Public Health and Primary Care Maastricht University Maastricht The Netherlands; ^5^ Kleijnen Systematic Reviews Ltd York UK; ^6^ Department of Clinical Epidemiology and Medical Technology Maastricht University Medical Centre Maastricht The Netherlands

## Abstract

**Background:**

Chronic pain is a major economic and social health problem. Up to 79% of chronic pain patients are unsatisfied with their pain management. Meeting patients’ expectations is likely to produce greater satisfaction with care. The challenge is to explore patients’ genuine expectations and needs. However, the term expectation encompasses several concepts and may concern different aspects of health‐care provision.

**Objective:**

This review aimed to systematically collect information on types and subject of patients’ expectations for chronic pain management.

**Search strategy:**

We searched for quantitative and qualitative studies. Because of the multidimensional character of the term “expectations,” the search included subject headings and free text words related to the concept of expectations.

**Data extraction and synthesis:**

A framework for understanding patients’ expectations was used to map types of expectations within structure, process or outcome of health care.

**Main results:**

Twenty‐three research papers met the inclusion criteria: 18 quantitative and five qualitative. This review found that assessment of patients’ expectations for treatment is mostly limited to outcome expectations (all 18 quantitative papers and four qualitative papers). Patients generally have high expectations regarding pain reduction after treatment, but expectations were higher when expressed as an ideal expectation (81‐93% relief) than as a predicted expectation (44‐64%).

**Discussion and conclusions:**

For health‐care providers, for pain management and for pain research purposes, the awareness that patients express different types of expectations is important. For shared decision making in clinical practice, it is important that predicted expectations of the patient are known to the treating physician and discussed.

Structure and process expectations are under‐represented in our findings. However, exploring and meeting patients’ expectations regarding structure, process and outcome aspects of pain management may increase patient satisfaction.

## 1 INTRODUCTION

In Europe, chronic non‐cancer pain of moderate to severe intensity occurs in approximately 19% of the adult population.^1^ The international society for the study of pain defines chronic non‐cancer pain (CNCP) as non‐malignant pain lasting 3 months or more, or as pain persisting beyond the time of expected healing. CNCP often lacks a clear associated pathology; prognosis is uncertain and varies considerably between patients and therefore can be difficult to treat.^2^ CNCP has a significant impact on health status, quality of life and daily activities such as paid work.^3^

A large proportion of CNCP patients lack adequate pain control.^3^, ^4^ Up to 79% of the CNCP patients believe that their pain is inadequately treated, and up to 43% of the patients report not receiving pain treatment at all.^5^ Given the subjective and objective burden of CNCP, the fact that a large majority of patients believe their pain is inadequately treated should alarm health‐care professionals and policymakers.^3^

Patients’ satisfaction with CNCP management can be seen as the end result of the match between expectations and subsequent experiences.^6^, ^7^, ^8^ From a theoretical conceptual point of view, patients’ expectations are viewed by some as the major determinant for satisfaction with health care. For example, according to the expectancy disconfirmation paradigm, satisfaction arises either from positive experiences disconfirming negative expectations. Dissatisfaction arises when negative experiences disconfirm positive expectations, or when negative experiences confirm negative expectations. Disconfirmation of expectations affects perceived quality of care, and hence satisfaction.^9^ Discrepancy between expectations and actual outcome portents lower satisfaction.^10^ Empirical evidence for the relation between expectations and satisfaction is for instance provided by Noble et al. They found that the fulfilment of patients’ satisfaction was primarily determined by patient expectations.^11^ Each patient with CNCP experiences pain in a highly individualized way, and each patient has different expectations, needs and goals. Therefore, pain management should also be customized, and understanding patients’ expectations is essential in shared decision making.^12^, ^13^ Meeting patients’ expectations should result in more consistency between the patients’ needs and health‐care delivery, and subsequently in greater satisfaction with care.^14^ Satisfaction with care might increase compliance, which, in turn, can improve pain management outcome.^15^

The challenge, however, is to identify the patients’ needs and expectations. The aim of this study was therefore to systematically explore the literature for information on patients’ expectations of CNCP management. As the term “expectations” comprises a broad range of concepts which can refer to several aspects of health‐care delivery, we start this review by defining and classifying expectations according to type of expectation and according to Donabedian's health‐care model of structure, process and outcome of care.

### 1.1 Categorizing patient expectations

Expectations are generally explained as “a strong belief that something will happen or be the case.”^16^ Related to anticipation, this implies that expectations are created and sustained by a cognitive process. An event, however, can be desired but not expected,^17^ for example “I desire to be cured after treatment but I expect only minor pain reduction.” Expectations, therefore, can also be expressed as desires, wishes and hopes.^8^ In contrast to beliefs, these primarily reflect a valuation mainly based on emotions, a perception that a given event is wished for. It is therefore important to distinguish the various types of definitions of the expectations used in research papers as these are sometimes lacking, and the reader is often left to guess whether the expectations described are hopes or ideals, or anticipated outcomes.

#### 1.1.1 Types of expectations

Thompson^7^ used the following terms to distinguish between types of expectations: ideal expectations, normative expectations, predicted expectations and unformed expectations. Unformed expectations are not articulated expectations.

1. Ideal expectations are visions, aspirations, needs, hopes and desires, related to the patient's views of the potential for a service.^7^
2. Normative expectations are expectations about what should or ought to happen, mostly derived from what users are told, or led to believe, or think that they ought (or to which one has a right) to receive from health services.^7^
3. Predicted expectations are beliefs about what will actually happen and are likely to result from personal experiences, reported experiences of others and other sources of knowledge such as in the media.^7^, ^8^

Kravitz^8^ distinguished between expectations as probabilities, that is the likelihood of future clinical occurrences, and expectations as values. Value expectations can be expressed as a hope or desire (what is wanted), necessity (what is perceived to be needed), entitlement (that which is owed or to which one has a right) and normative standards (that which should be).^8^ Kravitz^8^ described a dynamic model in which patients’ expectations are also defined according to content (i.e structure, process or outcome of care)^18^.

In this study, we consider “expectations as probabilities” and “predicted expectations” to reflect the same type of expectations. Throughout the study, we will refer to this as predicted expectations.

#### 1.1.2 Content: Structure, process and outcome of care

Patients may express their expectations regarding several aspects of health‐care delivery. The Donabedian's health‐care model provides a standard for examining health services and evaluating quality of health care and distinguishes between structure, process and outcome of care (SPO).^18^ Structure of care denotes the setting in which the care occurs, for example the characteristics of the building, accessibility, availability of therapeutic and diagnostic facilities. Process of care reflects what is actually done in care delivery and care coordination, for example provider characteristics, timing variables. It describes how the patient moves into, through and out of the health‐care system, and the services provided during the care episode. Outcome of care is about the effects of health care, for example, on the patient's health, functioning and quality of life. Research into the quality of health care shows a strong correlation between structure, process and outcome.^19^, ^20^

#### 1.1.3 Framework for understanding patient expectations

**Figure 1:**
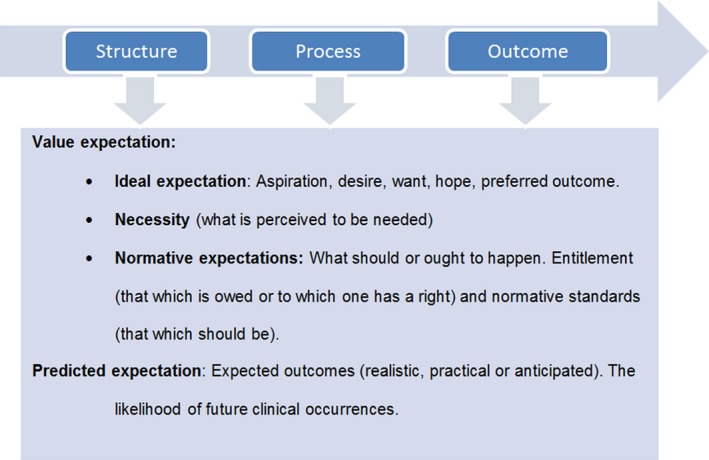
Framework for understanding expectations, composed using the study by Thompson AG, Sunol R, Kravitz RL, Donabedian A [Colour figure can be viewed at wileyonlinelibrary.com]

The term “expectations” is sometimes undefined, imprecise or multi‐interpretable; therefore, a conceptual framework is used to categorize the findings from the papers in this review (Figure 1). Expectations are classified according to the SPO model^18^ and the work of Thompson^7^ and Kravitz.^8^ Predicted expectations are cognitive, realistic and anticipated. Value expectations are attitudes, regulated by feelings, emotions and affections. The value expectations are divided according to Thompson^7^ into ideals, necessities and normative expectations (i.e entitlements/normative standards).

## 2 METHODS

### 2.1 Objectives

The main objective of this systematic review was to classify patients’ expectations regarding CNCP management according to the framework of understanding expectations (Figure 1). Secondary objective of this study was exploration of the subject of patients’ expectations.

### 2.2 Design

This systematic review explored expectations regarding CNCP management reported in quantitative, in qualitative, as well as in mixed methods research papers. The combination of quantitative, mixed methods and qualitative research was expected to generate a more complete and deeper insight than either method alone.

### 2.3 Eligibility criteria

Expectations of patients undergoing pain management continually change when experiences accumulate.^21^ Furthermore, patients with acute (less than 6 weeks), subacute (6‐12 weeks) and chronic (at least 3 months) pain exhibit different physiologies, courses and treatment responses.^22^ Therefore, it is highly likely that expectations regarding pain therapy differ before and after pain therapies and between (sub) acute and chronic patients. For this reason, this review was restricted to papers that described expectations regarding pain therapy before or during their pain management of chronic (>3 months) non‐cancer‐related pain. Pain management is defined as communication, evaluation, diagnosis and treatment, of all different types of CNCP.

Studies were considered eligible for review if they met the following inclusion criteria: (i) patients were questioned about expectations before or during CNCP management; (ii) the study population consisted of adult patients with chronic (≥3 months) non‐cancer‐related pain; (iii) measuring expectations was (one of) the objective(s) of the study, and the method for obtaining information on patients’ expectations was described. Exclusion criteria were as follows: (i) cancer‐related pain, (ii) pain duration of less than 3 months or (iii) pain duration not specified.

In case of inadequate or missing information about expectation(s) or definition of chronic pain, authors of the article were contacted for information. Studies were excluded from this review if multiple studies were identified, with overlap in study populations and findings. When this was the case, only the most appropriate (to our review objective) study was included to avoid potential duplication of data sets.

### 2.4 Search: Study selection

A literature search was performed for suitable articles published between 1990 and 2016, archived in Medline, PSYCHINFO, CINAHL and EMBASE. Owing to the broad range of concepts related to the term “expectations,” the search included subject headings and free text words connected to the construct expectations.^6^, ^7^, ^8^, ^17^ In Table 1, the search terms are given. Two authors (JG/PW) independently screened the titles, abstracts and keywords of all references identified by the literature search to determine whether they addressed the objective of our review. For potentially relevant articles, full‐text publications were retrieved. The bibliographies of all identified articles and relevant systematic reviews were screened for additional relevant studies.

**Table 1:** Medline search

Chronic pain MeSH	1 Chronic Pain/(7797)
	2 Pain, Intractable/(4246)
	3 1 or 2 (11924)
Pain MeSH combined with chronic free text terms	4 exp Pain/(337350)
	5 Pain Management/(34414)
	6 exp Analgesia/(39123)
	7 or/4‐6 (370490)
	8 (chronic$ or intractable or refractory or persistent$ or long term or longterm or sustained or longstanding or long standing or permanent$ or unremitting or unrelenting or unceasing or constant or constantly).ti,ab,ot. (2082083)
	9 7 and 8 (73595)
Chronic pain free text terms	10 ((chronic$ or intractable or refractory or persistent$ or long term or longterm or sustained or longstanding or long standing or permanent$ or unremitting or unrelenting or unceasing or constant or constantly) adj3 (pain or pains or painful$ or pained)).ti,ab,ot. (52779)
	11 ((chronic$ or intractable or refractory or persistent$ or long term or longterm or sustained or longstanding or long standing or permanent$ or unremitting or unrelenting or unceasing or constant or constantly) adj3 (hurt or hurting or hurts)).ti,ab,ot. (10)
	12 ((chronic$ or intractable or refractory or persistent$ or long term or longterm or sustained or longstanding or long standing or permanent$ or unremitting or unrelenting or unceasing or constant or constantly) adj3 (sore or soreness or tender$ or discomfort or ache$ or aching or agony)).ti,ab,ot. (881)
	13 ((chronic$ or intractable or refractory or persistent$ or long term or longterm or sustained or longstanding or long standing or permanent$ or unremitting or unrelenting or unceasing or constant or constantly) adj3 (nociception or nociperception or algiatry)).ti,ab,ot. (230)
	14 ((chronic$ or intractable or refractory or persistent$ or long term or longterm or sustained or longstanding or long standing or permanent$ or unremitting or unrelenting or unceasing or constant or constantly) adj3 (allodynia or alveolalgia or backache or causalgia or cephalalgia or cheiragra or chiragra or coxalgia or coxodynia or cystalgia or dorsalgia or dysmenorrh?ea or dyspareunia or dysuria or erythromelalgia or failed back surgery syndrome or fibromyalgia or gastralgia or headache$ or hepatalgia or intermittent claudication or ischialgia or lumbago or lumbalgia or lumbodynia or mastalgia or mastodynia or meralgia paresthetica or metatarsalgia or migraine$ or myalgia or neuralgia or odontalgia or odynophagia or orchalgia or otalgia or paroxysmal hemicrania or piriformis syndrome or piriformis muscle syndrome or pleuralgia or polymyalgia or prostatalgia or prostatodynia or psychalgia or rachialgia or radiculalgia or sciatica or SUNCT syndrome or toothache or vulvodynia)).ti,ab,ot. (8703)
	15 or/10‐14 (60583)
All chronic pain terms	16 3 or 9 or 15 (93343)
Patient expectation MeSH terms	17 Patient Acceptance of Health Care/(35853)
	18 Patient Participation/(20495)
	19 exp Patient Satisfaction/(71227)
	20 Self Efficacy/(14820)
	21 Physician‐Patient Relations/(64939)
	22 exp Attitude to Health/(341092)
	23 484/(165)
	24 motivation/(56062)
	25 decision making/(77220)
Patient expectation free text terms	26 ((patient$ or consumer$ or user or users or client$ or sufferer$ or person$ or people or adult$ or men or mens or man or mans or women$ or woman$) adj1 (ambition$ or aspiration$ or attitude$ or belief$ or believe$ or choice$ or concern$ or decision$ or demand$ or desire$ or drive or evaluation$ or expectation$ or experience$ or feeling$ or goal$ or hope$ or idea$ or impression$ or intention$ or judgment$ or motivation$ or motive$ or need or needs or opinion$ or perception$ or perspective$ or preference$ or reason$ or requirement$ or thought$ or value$ or view$ or wish$)).ti,ab,ot. (160415)
All patient expectation terms	27 or/17‐26 (624112)
Chronic pain terms combined with patient expectation terms	28 16 and 27 (7581)
Animal only terms	29 exp animals/not (exp animals/and humans/) (4301405)
Exclude animal only studies	30 28 not 29 (7553)
Limit publication year to 1990 to date	31 limit 30 to yearr=“1990‐2016” (7176)

### 2.5 Quality assessment

Quality assessment of the qualitative research papers was conducted by two independent reviewers(JG/CL) according to the Qualitative Assessment and Review Instrument (QARI).^23^ The QARI software was developed by the Joanna Briggs Institute (Australia) for the evaluation and synthesis of qualitative research articles. This quality appraisal tool is a standardized 10‐criteria checklist for two independent reviewers and assesses bias in relation internal validity to, for example, congruence between research methodology, philosophical perspective, methods used to collect data, analyse the data and for interpretation of the data.

Assessment of the quantitative and mixed methods research papers was performed with the Mixed Method Appraisal Tool (MMAT).^24^ This appraisal tool was developed for the quality assessment in reviews that include quantitative, qualitative and mixed methods studies. With this instrument, it is possible to judge each paper in relation to its methodological domain.

### 2.6 Data collection, extraction and synthesis

Extraction of findings of the qualitative papers was performed using the Qualitative Assessment and Review Instrument (QARI). (Joanna Briggs Institute Reviewers Manual 2014). An expectation finding was defined as a theme, metaphor or data by the author supported by quotes from the patient, fieldwork observations or other data. Only unequivocal and credible findings were considered for evaluation; these are findings that are matter of fact, directly reported/observed and not open to challenge.

To categorize patients’ expectations, a metasynthesis of the papers is presented in a tabular summary, using the framework of Figure 1. First, we categorized health care into structure, process and outcome of care. Within each health‐care category, two major types of expectation were classified: predicted and value expectations. (Introduction chapter 1.1) Value expectations were subdivided into ideals (hopes, wishes, desires), necessities (needs) and normative expectations (entitlements).

Mixed methods studies in this systematic review were evaluated as quantitative papers because the analyses were quantitative, although the assessment often was mostly qualitatively performed. Three authors JG/CD/PW independently categorized the types of expectations. Differences in categorization were discussed and solved in a consensus meeting.

## 3 RESULTS

### 3.1 Study selection

**Figure 2:**
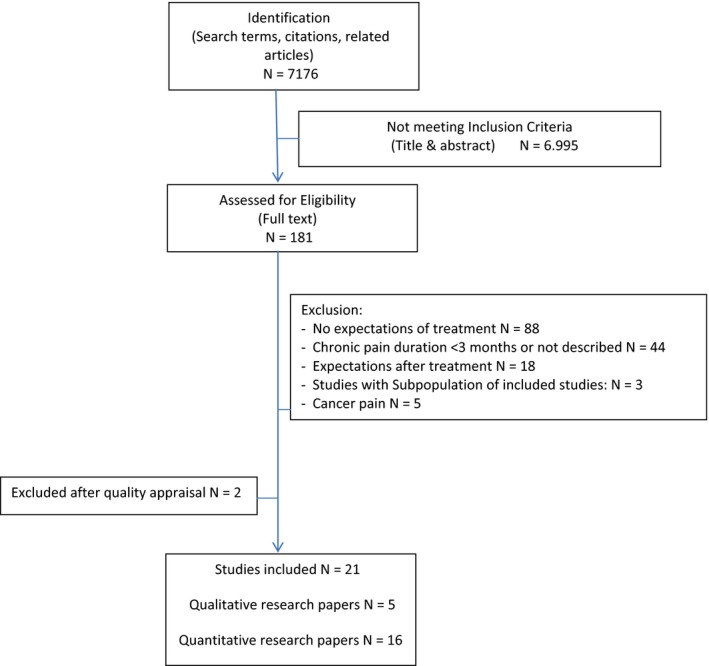
Flow diagram of the literature search process [Colour figure can be viewed at wileyonlinelibrary.com]

Figure 2 shows a flow diagram of the study selection, procedure and results. The full text of 172 papers was assessed according to the inclusion and exclusion criteria. The most frequent reason for exclusion in this review was when papers did not describe pain management expectations but for instance experiences. Furthermore, in a substantial number of papers, the research population included acute and subacute pain patients. If results were not presented separately for the subgroup of chronic pain patients, these papers were excluded from the review. In 13 papers, the definition of “chronic pain” used for selecting the research population was not clear. In these cases, the authors were contacted for information; based on their response, another three papers were included. Of the 23 remaining included studies, 18 were quantitative and five were qualitative studies.

### 3.2 Study characteristics

Table 2 shows the characteristics of the included studies. In most quantitative studies (N=18), a self‐constructed questionnaire^25^, ^26^, ^27^, ^28^, ^29^, ^30^, ^31^, ^32^, ^33^ was used; six studies used a validated questionnaire.^34^, ^35^, ^36^, ^37^, ^38^, ^39^ Most studies (N=12) were conducted in the USA, seven in Europe, two in Australia and two in Asia. Research aims and management settings were diverse. Chronic spinal pain was the most studied type of pain (11 studies).

**Table 2:** Study design and characteristics

Study	Study Design, Measurement^a^	N	Research Aims^a^	Country	Treatment/setting	Type of pain
Boonstra et al. 2011^25^	Quantitative, self‐constructed questionnaire with consensus study	616	To identify differences between males and female in expectations about goal achievement.	The Nether‐lands	Rehabilitation/General rehabilitation centre	Chronic musculoskeletal pain >3 months
Casaret et al. 2001^44^	Mixed method; structured interview, quantitative analysis	37	To define endpoints of pain research that are important to patients with chronic pain.	USA	Medication opioids/Anaesthesia pain clinic	Chronic pain >6 months
Eaves et al. 2015^46^	Qualitative, semistructured interviews	64	To determine how participants’ expectations of treatment change over the course of a therapy.	USA	Complementary and alternative medicine/CAM practitioners	Chronic low back pain >3 months
Groeneveld et al. 2008^34^	Quantitative, Joint Replacement Expectations Survey (JRES).	909	To measure racial differences in expectations of improvement of quality of life after Joint Replacement Surgery	USA	Joint replacement surgery/Primary care at 2 veterans affairs medical centres	Chronic knee or hip pain ≥ 6 months
Hazard et al. 2012^26^	Quantitative, self‐constructed questionnaire	82	To examine the expectations about goal achievement	USA	Functional restoration program/Spine centre	Chronic back and/or neck pain > 3 months
Hsu et al. 2014^47^	Quality, semistructured interviews	64	To examine the outcome expectations of patients prior to receiving CAM therapies.	USA	Complementary and alternative medicine/CAM practitioners	Chronic low back pain> 3 months
Iversen et al. 1998^27^	Quantitative, self‐constructed questionnaire	257	To determine how patient expectations interact to predict post‐operative outcomes.	USA	Lumbar spinal stenosis surgery/Hospitals	Back, buttock and/or leg pain due to Lumbar Spinal Stenosis
Linde et al. 2007^28^	Quantitative, self‐constructed questionnaire	864	To examine the association of expectations with response to treatment	Germany	Acupuncture/CAM practitioners	Chronic pain ≥12 months
Nielsen et al. 2013^42^	Qualitative, unstructured narrative interviews	20	To examine the health‐care experiences of people with chronic pain	Australia	Primary care	Chronic pain ≥6 months
O'Brien et al. 2010^35^	Quantitative, patient‐centred outcomes questionnaire (PCOQ)	300	To define patient‐determined success criteria for pain treatment	USA	Pain treatment/Rheumatology outpatient clinic	Fibromyalgia and chronic back pain >3 months
Petrie et al. 2005^41^	Mixed method; structured interview, quantitative analysis	77	To examine patients’ expectations of their first outpatient pain clinic consultation	USA	Pain management/First visit Pain clinic	Chronic pain ≥6 months
Sanderson et al. 2012^36^	Quantitative, patient‐centred outcomes questionnaire (PCOQ)	47	To determine pain treatment outcomes that patients consider clinically meaningful	USA	Chronic analgesic management program/Pain clinic	Chronic low back pain >3 months
Sherman et al. 2010^29^	Quantitative, self‐constructed questionnaire	477	To determine whether patients’ expectations for acupuncture predict short and long‐term treatment outcomes	USA	Acupuncture/Complementary and alternative medicine practitioners	Chronic low back pain >3 months
Smeets et al. 2008^37^	Quantitative, Credibility/Expectancy Questionnaire (CEQ),	167	To examine the association between treatment credibility/expectancy and patient characteristics, and outcome.	The Netherlands	Rehabilitation/Outdoor rehabilitation centre	Chronic non‐specific low back pain >6 months
Stutts et al. 2009^38^	Quantitative, patient‐centred outcomes questionnaire (PCOQ)	52	To define treatment success from the pain patient perspective.	USA	Rheumatology clinic	Fibromyalgia > 3 months
Thorne& Morley 2009^45^	Mixed method, structured verbal question, multidimensional pain inventory (MPI), Brief Pain Inventory (BPI)	78	To investigate the patient‐determined criteria for the magnitude of change necessary to achieve an ‘acceptable outcome’.	UK	Chronic pain out‐patient clinic	Chronic pain > 6 months
Toye & Barker 2012^43^	Qualitative, in‐depth interviews	20	To present a conceptual analysis of patients’ experience of general practice in relation to their persistent non‐specific low back pain.	UK	Physiotherapy/Chronic pain out‐patient clinic	Persistent non‐specific low back pain >3 months
Toyone et al. 2005^30^	Quantitative, self‐constructed questionnaire	49	To determine the patient expectations for spine surgery (lumbar spinal stenosis).	Japan	Surgery/Department of orthopaedic surgery	Lumbar spinal stenosis >3 months
Triva et al. 2013^31^	Quantitative, self‐constructed questionnaire	100	To study the association between patient satisfaction and accessibility of health‐care services.	Croatia	Pharmacological; physical therapy; acupuncture/University hospital pain clinic	Chronic pain >6 months
Turner et al. 2002^32^	Quantitative, self‐constructed questionnaire	84	To examine the association of expectations of pain relief with actual pain relief.	USA	Pharmacology/Department of rehabilitation medicine	Spinal cord injury: chronic pain >3 months
Wainwright et al. 2014^40^	Qualitative, nominal group technique	6	To engage stakeholders in the development of a community‐based chronic pain management service and identify their different agendas for service design and delivery	UK	Contextual cognitive behavioral therapy/Community based pain management service	Chronic pain >3 months
Yelland & Schluter 2006^33^	Quantitative, self‐constructed questionnaire	110	To describe patients’ perceptions of minimum worthwhile and desired reductions in pain and disability	Australia	Injections and exercises/University general practice clinic	Chronic low back pain >6 months
Yi et al. 2014^39^	Quantitative, patient‐centred outcomes questionnaire (PCOQ)	50	To examine the relationship of the patient's criteria of successful treatment to emotional factors.	Korea	Rehabilitation/Department of rehabilitation medicine	Musculoskeletal pain >6 months

a Regarding expectations.

### 3.3 Quality Appraisal

The quality of the studies was appraised using the MATT^24^ and QARI appraisal tools, Tables 3a,b for, respectively, quantitative (including mixed methods studies) and qualitative studies.

**Table 3:** Critical appraisal results for the quantitative studies using the (a) Mixed Method Appraisal Tool (MMAT)^24^and (b) JBI‐QARI Appraisal checklist^23^

^a^Study, Year	1.1	1.2	1.3	1.4	4.1	4.2	4.3	4.4	5.1	5.2	5.3	%
Mixed methods studies												
Casaret et al. 2001^44^	Y	Y	N	N	Y	Y	N	N	Y	Y	N	54
Petrie et al. 2005^41^	Y	N	N	N	Y	Y	N	Y	Y	Y	N	54
Thorne& Morley 2009^45^	Y	Y	N	N	Y	Y	N	Y	Y	U	N	54
Quantitative studies												
Boonstra et al. 2011^25^					Y	Y	Y	N				75
Groeneveld et al. 2008^34^					Y	Y	Y	U				75
Hazard et al. 2012^26^					Y	Y	N	U				50
Iversen et al. 1998^27^					Y	Y	N	Y				75
Linde et al. 2007^28^					Y	Y	N	U				50
O'Brien et al. 2010^35^					Y	Y	Y	U				75
Sanderson et al. 2012^36^					Y	N	Y	U				50
Sherman et al. 2010^29^					Y	Y	N	U				50
Smeets et al. 2008^37^					Y	Y	Y	Y				100
Stutts et al. 2009^38^					Y	N	Y	U				50
Toyone et al. 2005^30^					Y	Y	N	Y				75
Triva et al. 2013^31^					Y	N	N	Y				50
Turner et al. 2002^32^					Y	U	N	Y				50
Yelland & Schluter 2006^33^					Y	Y	N	Y				75
Yi, T. I., et al. 2014^39^					Y	U	Y	Y				75

^b^Study, Year	Q1	Q2	Q3	Q4	Q5	Q6	Q7	Q8	Q9	Q10	%	
Eaves et al., 2015^46^	U	Y	Y	Y	Y	N	N	Y	Y	Y	70	
Hsu et al., 2014^47^	N	Y	Y	Y	Y	N	N	Y	Y	Y	70	
Nielsen et al., 2013^42^	N	Y	Y	Y	Y	N	N	Y	Y	Y	70	
Toye et al., 2012^43^	Y	Y	Y	Y	Y	Y	N	Y	Y	Y	90	
Wainwright et al. 2014^40^	Y	Y	Y	Y	Y	U	U	Y	Y	Y	80	

Y, yes; N, no; U, unclear.

As our interest only related to pain management expectations, and these were collected mostly at baseline, all the quantitative and mixed methods papers were appraised as descriptive studies. On item 4.3 (“Are measurements appropriate”), for 11 of the 18 quantitative papers, the scores were zero because these studies used self‐constructed questionnaires without validation. The quality of the quantitative papers was good to excellent with ten papers reaching 50‐54%, seven 75% and one paper scoring 100%.

Most qualitative studies (Table 3b) scored low on item 1: “There is congruity between the stated philosophical perspective and the research methodology.” Almost all studies scored zero on items 6 and 7, that is 6) “There is a statement locating the researcher culturally and theoretically” and 7) “The influence of the researcher on the research and vice versa is addressed.” However, the overall quality of the accepted qualitative papers was rather high, scores ranged from 70% up to 90%; therefore, all papers were included in this review.

### 3.4 Findings

#### 3.4.1 Categorization of expectations according to the framework

Table 4 shows the results of the categorization by type of expectation and content (SPO) of care delivery. Two papers studied structure expectations, four process expectations and 21 outcome expectations. All quantitative papers (N=18) described outcome expectations. One‐third of the quantitative papers described both value and predicted expectations.

**Table 4:** Types of expectations found in research papers categorized within structure, process and outcome of care

Type expectation	Structure	Process	Outcome	Total
	N papers	N papers	N papers	
Quantitative	1	1	18	18
Value (only)	1 (0)	1 (0)	10 (5)	12
Predicted (only)	0 (0)	1 (0)	13 (8)	14
Both Value & Predicted	0	1	5	6
Qualitative	1	3	3	5
Value (only)	1 (1)	3 (2)	3 (2)	7
Predicted (only)	0 (0)	1 (0)	2 (0)	3
Both Value & Predicted	0	1	2	3
Total Sum	2	4	21	23

Only, restricted to this type of expectation.

Qualitative studies described more frequently (N=7) value expectations. Sixty per cent of qualitative papers described both value and predicted expectations.

##### 3.4.1.1 Structure expectations

Table 5 shows types of patients’ expectations found in quantitative studies, and Table 6 shows expectations found in qualitative studies.

**Table 5:** Findings categorized by health‐care process and subdivided by types of expectations. Quantitative Findings

Study	Outcome expectations				Structure				Process			
	Value			Predicted(P)	Value			P	Value			P
	Ideal(Id)	Necessity(Ne)	Normative (N)		Id	Ne	N		Id	N	N	
Boonstra et al. 2011^25^				Pain Reduction, Pain Cure, Improvement physical, psychological, Daily Social Activity, Coping, Diagnosis, Work, Sleep, Medication								
Groeneveld et al. 2008^34^				Pain Reduction, Improvement physical, psychological, Daily Social Activity, Sleep, Medication, SexA, Work, QOL								
Hazard et al. 2012^26^				Pain Reduction, Improvement physical, Daily Social Activity, Work								
Linde et al. 2007^28^				Complaints cure/relief								
Iversen et al. 1998^27^	Pain Reduction, Pain Cure, Improvement physical, psychological, Daily Social Activity											
O'Brien et al. 2010^35^	Pain Reduction LBP 93%/Fybr 80%, Improvement fatigue, psychological, Daily Social Activity	Pain Reduction LBP 58%/Fybr 55%, Improvement fatigue, psychological, Daily Social Activity		Pain Reduction LBP 52%/Fybr 52%, Improvement fatigue, psychological, Daily Social Activity								
Sanderson et al. 2012^36^	Pain Reduction, Improvement fatigue, psychological, Daily Social Activity	Pain Reduction 65%Improvement fatigue, psychological, Daily Social Activity		Pain Reduction, Improvement fatigue, psychological, Daily Social Activity								
Sherman et al. 2010^29^	Pain Reduction											
Smeets et al. 2008^37^				Improvement physical								
Stutts et al. 2009^38^	Pain Reduction Facial 86%/Fybr 81%,Improvement fatigue, psychological, Daily Social Activity	Pain Reduction Facial 62%/Fybr 56%,Improvement physical, psychological, Daily Social Activity		Pain Reduction Facial 64%/Fybr 51%, Improvement physical, psychological, Daily Social Activity								
Toyone et al. 2005^30^				Pain Reduction, Improvement physical, Complaints, Daily Social Activity, Complications								
Triva et al. 2013^31^				Pain Reduction, Pain Cure		Eff						
Turner et al. 2002^32^				Pain Reduction 71%								
Yelland & Schluter 2006^33^	Pain Reduction 86%Improvement physical, Daily Social Activity	Pain Reduction 28%Improvement physical, Daily Social Activity										
Yi,T. I. et al. 2014^39^	Pain Reduction, Improvement fatigue, psychological, Daily Social Activity	Pain Reduction 74%Improvement fatigue, psychological, Daily Social Activity		Pain Reduction 44% Improvement fatigue, psychological, Daily Social Activity								
Mixed methods												
Casaret et al. 2001^44^	Pain Reduction, Improvement psychological, Daily Social Activity, SexA Medication, SE,											
Petrie et al. 2005^41^	Pain Reduction, Pain Cure, Medication		Pain Reduction, No change	Pain Reduction, Pain Cure, Coping, Medication					Validation, Con	DE	Diagnosis, ThO, DE	Diagnosis
Thorne & Morley 2009^45^		Pain Reduction 52%, Improvement physical, Daily Social Activity										

Coping, coping with pain; Con, thorough consultation including physical examination and tests; DE, disease explanation; Diagnosis, firm diagnosis; Eff, efficient flow through the system; Fybr, fibromyalgia; LBP, low back pain; Medication, management of medication; QOL= improvement quality of life; SexA, improvement sexual activity; SE, decreased side effects of therapy; ThO, therapy options; Work, improvement coping with work.

**Table 6:** Findings out of qualitative papers categorized by health‐care process and subdivided by types of expectations

Quote	Outcome				Structure				Process			
	Value			P	Value			P	Value			P
	Id	Ne	N		Id	Ne	N		Id	Ne	N	
I'm hoping that long term that this will lessen my pain and give me a better quality of life That's what I'm hoping for But I'm not going in with an expectation that this is what's going to happen. Study Hsu,p4^47^	PainRQOL			No Change								
Oh, I think realistically, I don't think it'll change much I would hope that it would help, I hope I would have some reduction in the amount of pain that I have, especially at this moment. Study Hsu,p4^47^	PainR			No Change								
I guess my hope would be that the uncomfort in my back is gone but honestly I try not to have any expectations Because if it doesn't work then that's not very much fun, to have a bunch of expectations and it doesn't work. Study Hsu,p4^47^	Comfort			No Change								
… as far as what I expected, that was totally different, I didn't expect anything, I expected nothing, nothing one way or another. Study Hsu,p5^47^				No Change								
My hope would be for my back pain to be relieved or maybe eradicated that would be great My expectation is that it could be improved, but not necessarily eradicated. Study Hsu,p5^47^	PainR											
PainC			PainR									
I think that there's something out there for me that'll work I don't just want to accept the fact that I'm in pain I don't‐ and I don't want to cover it up with drugs I want it to be fixed Something is wrong if I'm hurting and I want it fixed. Study Hsu,p5^47^			PainC									
I just want to be able to manage it and decrease the amount of time that it hurts, the duration of the hurt. Study Hsu,p5^47^	PainR											
Well I would hope‐the bottom line is that I want to be relieved of the pain that I have I would say I don't have expectations beyond the current pain. Study Hsu,p6^47^	PainC			No Change								
I didn't think it would eliminate my pain, I just thought it would help the healing and help, you know, me be more comfortable, but it wouldn't make it go away. Study Hsu,p6^47^				PainRComf								
Oh, I would do a lot more walking and a lot more physical things and more yard work, more being with my dogs. I have Basset Hounds so they're all short. You mostly need to get on the floor with them. And, you know, and I can get down on the floor, it's getting back up that just brings tears to my eyes and I want to be able to do that. Study Hsu,p6^47^	Phys											
I'm hoping that just little things, like I can do the walk around the little water pond with my grandkids… I mean I don't want to go run a marathon, I don't think I'll do that anytime soon, I never ran before I got sick, you know what I mean? Just the little things, day to day, being able to vacuum, and clean the bathrooms on the same day, I can't do that right now, I just want to be able to do, to complete that task, ‘… Study Hsu,p6^47^	PhysDSA											
I'm just hoping that it will provide me with a little more strength to support my back so that I can do things like vacuum the house or just whatever without just I guess decreasing the risk of triggering the back pain from coming back as often as it has been lately. Study Hsu,p6^47^	PainRPhysDSA											
I think it would just help the overall not—or trying not—to slide into being depressed about it. Not have to use up so much strength and energy just to marshal all my horses to carry on even though I hurt so much. Study Hsu,p6^47^	Phys	Ps										
You know, I think that my life would improve because I am so irritable, it's just kind of bad It makes me sad that, yeah, it's really depressing sometimes, I mean I normally wouldn't be, and so I think I would just be in a more peaceful place. Study Hsu,p6^47^	Psych											
I was hoping that it could basically allow me to restore my daily routine and quality of life as it was before the acute episode happened. Study Hsu,p6^47^	DSAQOL											
Bottom line is that I want to be relieved of the pain that I have I would say I don't have expectations beyond the current pain In other words I'm not going into this thinking that as a result of the treatment I'm no longer going to have back pain. Eaves,p5^46^	PainR			No Change								
Oh, I thought it would definitely get better I was really assuming that I would have, you know, less pain and that maybe it would take a number of treatments, but that eventually it would help alleviate the problem…I was hoping [it would cure] Eaves,p5^46^	PainC			PainR								
I think it will give me tools to kind of control it, more tools to enable my body to be aware of some of the different muscles or areas or maybe things I shouldn't do to it, to help control the pain or also learn different things that maybe can relieve it [so]…it's not causing the pain Eaves,p5^46^				PainRCop								
I don't [expect my life to change], I'm not an optimist by nature, certainly lost my optimism through this whole thing, I really don't expect much. Eaves,p7^46^				No Change								
I probably expected something a bit more thorough ………I would have expected an X‐ray, a blood test, a something. Toye, p78^43^											Cons	
…you don't expect people to swoon all over you, but just to say, ‘I understand, I think’, and just look as if he is willing to want to help…Toye,p78^43^											Valid	
…when you go to the GP and say, I have got back pain, really all they can do is send you off and refer you. Toye,p79^43^												Ref
A GP is exactly what it is, a general practitioner, he is not a specialist in bones or whatever, but you really do need an ‘expert’, in inverted commas I wanted them to refer me so that I could talk to an expert. Toye,p79^43^											Ref	
‘If only they could tell me what it is!’: searching for a diagnosis and cure Nielsen,pC^42^	Cure								Diagn			
All participants continued to hope for an effective resolution of their pain, although many had stopped actively searching for this in the medical system Nielsen,pC^42^	PainC											
Many participants commented on their desire to be listened to and believed by health care practitioners Mat made a plea for doctors to talk to people with chronic pain ‘like they're people, not an X‐ray walking through the door’ Nielsen,pD^42^									Valid		Valid	
Patients or fellow‐sufferers should be involved in the delivery of the service A key motive was the desire to evoke deep empathy from the providers of the intervention ‘I don't think it really matters per se who would run the session I rather think that somebody that's actually suffered from chronic pain and has had training to stand up there with your GP or your nurse or whoever and has actually experienced what you're going through to one degree or another […] but I think that it's very important because you can be spoken to and you think “you haven't got a clue, you don't know what it's like” That would be my main issue’ Wainwright, p785^40^					Pat						Valid	
..it's more about emotional support than technical support […] I just feel the group sessions can be more effective sometimes, talking to other people, you do feel very alone and just understanding there are other people that have this. Wainwright, p 786^40^					Pat						Valid	
If the people that are dealing with you don't understand what it's like for a person in chronic pain…So you need somebody in that position that is going to understand why you feel like this. Wainwright, p 786^40^										Val		
Regular contact really, because sometimes you can feel you're alone […]so just someone at the end of a telephone some days when you are particularly bad. Wainwright, p787^40^						SR						
When you hit a low, it would be nice to duck back into the service, ring them up and say “look, you know, it's flared up again, can I come back in?”‘ Wainwright, p787^40^					SR							
Yeah it is being treated like an individual but it's also looking at the patient as a whole […]rather than, “well you fit into that box so you're going to have all that treatment that goes with that condition”[…] physiotherapy, massage, helps your body cope and your mind then copes better. Wainwright, p788									CopinValid			
Accessibility Venue: ‘sure you can get there’[…] it's not how far, it's “can I drive? Can I park close? How much pain inducing movement have I got to go through?”’ Wainwright, p788^40^						Ac						
Accessibility opening times: ‘Unfortunately some people are better in a morning, some people like myself are better in an afternoon If you're working how on earth are you going to do a 6 week course if it's mornings or afternoons’ Wainwright, p789^40^						Ac						

P, predicted expectations; Id, ideal expectations; Ne, expectations expressed as necessity; N, normative expectations; PainR, Pain Reduction/Relief; PainC, Pain Cure; Phys, Improvement Physical; Psych, Improvement Psychological wellbeing; QOL, Improvement quality of life; DSA, Improvement Daily Social Activity; Ac, accessibility; Con, thorough consultation; Diagn, firm diagnosis; Cop, coping; Pat, patient involvement; Ref, referral; SR, support reachable; Valid, validation of the pain problem.

Only value expectations were found regarding structure of care; these value expectations were expressed as ideals or necessities. Patients expressed the desirability of fellow patient involvement in a chronic pain management service, mostly to support the patients in their contact with the professionals and achieve validation of their pain problem^40^ (Table 5). Further structure expectations were desirability of efficient flow of patients through the system (Table 5) and need for accessibility, for example parking places nearby and variable opening times (Table 6).

##### 3.4.1.2 Process expectations

Research addressing expectations regarding process of care was found in one quantitative^41^ (Table 5) and in three qualitative studies^40^, ^42^, ^43^ (Table 6). All studies reported value expectations of which two also showed predicted expectations. Regarding process expectations, explanation or improved understanding of the pain problem was expressed as a necessity; validation or acknowledgement of the pain problem was expressed mostly as a normative expectation, and to get a proper diagnosis was stated as an ideal expectation. Getting a thorough consultation or referral from the GP to a specialist was once expressed as a predicted expectation and once as a normative expectation.

##### 3.4.1.3 Outcome expectations

Most studies, all 18 quantitative and three (of five) qualitative, reported outcome expectations, of which 15 papers showed outcome expectations only. Fifteen papers reported predicted outcome expectations and 13 studied value expectations.

Almost all of the quantitative studies investigated predicted expectations in terms of pain management goals, like expected outcome. Four studies focused on value expectations, for example desired, disappointing, worthwhile or outcome needed to consider the pain management a success.^27^, ^29^, ^33^, ^44^

Four papers studied expected pain relief before pain treatment and related this to the pain reduction acquired after treatment. All showed that patients expected a substantially larger reduction in pain from the treatment than they attained.^30^, ^36^, ^38^, ^45^ For instance, patients needed a mean 50.9 (scale 1‐100) reduction and only attained 11.9.^36^ Whenever available in the papers, the expected levels of pain reduction by type of outcome expectation are included in Table 5a. In all quantitative studies, in which the ideal pain relief and expected pain relief were assessed separately, the results showed discrepancies between desired, needed and predicted pain relief. The expected pain relief was notably less than the stated needed and desired pain relief.

The qualitative studies (Table 6) also showed great discrepancy between the desired and the expected outcome: Patients often expressed a want or a need for pain relief or pain cure but predicted substantial less pain relief or no pain reduction at all.^46^, ^47^

Within each setting of care delivery, that is primary care, CAM, surgery, rehabilitation, pain centres, most CNCP patients expected pain relief; however, some patients did not expect pain relief but expressed the desire and need for physical improvement and being able to walk with the grandkids for instance, or do daily living chores without limitations. Some patients expressed the need to learn to cope with the CNCP, or to learn tools for better control of the complaints.

## 4 DISCUSSION

In this review, we systematically searched for quantitative and qualitative studies addressing expectations of chronic pain patients regarding CNCP management and categorized expectations according to the type of expectation and Donabedian's health‐care model of structure, process and outcome.

This review found that assessment of CNCP patients’ expectations for pain management is mostly limited to outcome expectations. Furthermore, we found that patients answer differently to questions pertaining predicted expectations than to questions about ideal expectations. Patients’ ideal expectations are higher than their predicted expectations; some patients hope for, or desire, a full cure, but predict to gain little or nothing from pain management. This discrepancy between ideal and predicted expectations could be due to negative experiences in the past, or it could be that patients lower their expectations as a way to avoid disappointment.^48^ Another explanation, which logically follows from Thompson^7^, is that the terms “hope” and “desire” actually mean something else to patients than the term “expectation,” irrespective of their previous experiences. In that case, it could well be that patients are in the process of accepting the pain and consequently suffer less pain and thus expect (predicted expectation) less gain from pain management than they would perceive as ideal (value expectation).^49^, ^50^ Empirical studies have demonstrated a positive association between acceptance and successful adaptation to chronic pain.^50^

Results of the papers in our review showed that overall CNCP patients’ expectations of pain reduction after treatment are high. This is most certainly true for the ideal expectations. This alone can lead to dissatisfaction with pain management. Improvement of pain management could be the answer (e.g preventing patients not receiving pain treatment, development of better pain therapies, incorporation of patients’ expectations into shared decision making and individualized pain management). However, it is known that often, even if the clinical outcome expectations are met, some patients are still dissatisfied.^30^ Thus, focusing on improvement of outcome alone does not seem to be the answer, for outcome of care is also dependent on structure and process of care.^51^ There is some evidence for CNCP patients, who mostly have extensive experience with health care, that structure and process expectations are even stronger predictors of pain management satisfaction.^52^, ^53^ Despite aforementioned, the results of this review show that only few studies have addressed CNCP patients’ expectations regarding structure and process aspects of pain management.

Our results show that the expectations as expressed by the patients depended on which way the questions were asked. For instance, when asking for desired (value expectation) levels of pain after treatment, patients reported to wish for up to 98% pain relief versus when asked “what to expect the treatment to do” (predicted expectation), patients reported far more realistic pain reductions of 50%. Therefore, it is highly probable that the relationship between “value” expectations and outcome differs from the relation between “predicted” expectations and outcome. Six studies in this review demonstrated this by assessing the relation between outcome and expectations.^28^, ^29^, ^32^, ^33^, ^36^, ^37^ A significant association between expectations and outcome was found in three papers that studied predicted expectations: Higher expectations of outcome resulted in more improvement.^28^, ^32^, ^37^ In contrast, the other three studies that assessed the association between value expectations and outcome did not find an association with outcome.^29^, ^33^, ^36^ Therefore, it seems that not only for pain management but also for research purposes the type of expectation assessed should be clear.

We found that most quantitative papers did not use validated expectation scales. This could be due to the fact that applied research into patients’ expectations is still in its infancy. Developing and validating expectation scales that comprise structure, process and outcome expectations as well as the different types of expectations would be helpful for shared decision making and could provide a useful tool for expectation management during pain therapies.

The incorporation of findings into a predefined expectation framework can be seen as a strength of this systematic review. Working with a framework to categorize types of expectations found in the papers leads to a better understanding of the broad concept and terms related to “expectations.” However, the original papers did not always provide a typology of expectations, leaving this open to our interpretation. Specifically, within value expectations, distinguishing between necessities and normative expectations was particularly challenging. The categorization was therefore performed by three authors independently (JG, PW, CD), and differences were discussed until consensus was reached.

Another strength of this systematic review is the combination of quantitative, mixed methods and qualitative studies. Qualitative findings added context or explanatory powers to the quantitative data, whereas quantitative data were useful to assess the size of the topic of interest. Furthermore, we found that qualitative findings provided more information about expectations regarding process and structure of care. However, some qualitative studies also restricted themselves to asking focused questions and explored or reported outcome expectations only.^46^, ^47^

For health‐care providers, for pain management and for pain research purposes, the awareness that patients express different types of expectations is important. For health‐care providers, it points at the importance of asking the right question about expectations in shared decision making and in expectation management. A validated questionnaire that incorporates all types of expectations that are assessed before the first consultation would be a useful tool to ensure manageable answers from the patient and discover genuine needs that should be incorporated into the pain treatment plan. Furthermore, this asset could also help in shared decision making to discover and discuss unrealistic expectations for treatment so as to avoid disappointment and dissatisfaction with care.

Health‐care providers and policymakers should grasp the opportunity to improve on structure, process and outcome of care and thereby attain higher patient satisfaction by better meeting patients’ expectations.

### 4.1 Clinical implications

This systematic review showed that little information could be found about structure and process expectations of CNCP patients. We like to point out that this could be a lost opportunity to derive higher patient’ satisfaction for CNCP management. It is known that structure and process components of care can influence pain patient’ satisfaction.^51^, ^52^ For instance, a strong positive association was found between higher numbers of physicians and nurses and patient’ satisfaction with the health‐care system.

Understanding the expectations and needs of patients is essential in shared decision making.^13^ Therefore, it is important to differentiate between the types of expectations. In particular, the difference between value and predicted expectations is important in clinical practice. Value expectations are ideals, and predicted expectations are the more realistic expectations. This review gives an indication that the association between high expectations and a better outcome is present when assessing predicted (i.e more realistic) expectations. In contrast, no association was found between high ideal expectations and better outcome. Patients’ predicted expectations for a specific treatment can be altered by information from the professional about the evidence for potential benefits and harms of a treatment for an individual patient. Management of expectations before and during pain management could be an important contribution to patients’ satisfaction by lowering predicted expectations that are too high or heighten predicted expectations that are too low.

Differentiating between types of expectations could also be important if patients are in the process of accepting the pain better and consequently struggling less with the pain.^50^ The pain management challenge should be to provide a personalized pain management programme without obstructing the patient's pain acceptance process. In shared decision making, it is likely that the process of pain acceptance is supported if predominantly predicted expectations are discussed.
